# An observational cohort study on impact, dimensions and outcome of perceived fatigue in adult 5q-spinal muscular atrophy patients receiving nusinersen treatment

**DOI:** 10.1007/s00415-020-10227-5

**Published:** 2020-10-07

**Authors:** Camilla Binz, Olivia Schreiber-Katz, Mareike Kumpe, Gresa Ranxha, Hannah Siegler, Gary Wieselmann, Susanne Petri, Alma Osmanovic

**Affiliations:** Department of Neurology, Hannover Medical School, Carl-Neuberg-Strasse 1, 30625 Hannover, Germany

**Keywords:** Fatigue, Fatigue severity scale (FSS), Multidimensional fatigue inventory (MFI), Spinal muscular atrophy (SMA), Nusinersen, Patient-reported outcome measure (PRO)

## Abstract

**Background:**

Efficacy of nusinersen in adult 5q-spinal muscular atrophy (SMA) patients regarding motor function has recently been demonstrated. However, additional outcome measures are needed to capture non-motor improvements. Fatigue is a common and disabling symptom in neurologic diseases, but little is known about its frequency, characteristics and associated factors in SMA.

**Objective:**

To characterize fatigue in SMA patients receiving nusinersen, identify associated factors and evaluate fatigue as potential patient-reported outcome measure (PRO).

**Methods:**

We assessed fatigue in adults with genetically confirmed 5q-SMA in a prospective longitudinal monocentric study using the Fatigue Severity Scale (FSS) and the Multidimensional Fatigue Inventory (MFI). Factors associated with fatigue including health-related quality of life (HRQOL) were evaluated.

**Results:**

75% of participants were abnormally fatigued with highest scores in the dimensions* physical*, followed by *general fatigue* and *reduced activity*. 53% agreed that fatigue was among their three most disabling symptoms. *Reduced activity* was reported more extensively by participants with ≥ 4 copies of the *survival of motor neuron 2* gene and better motor function. *General* and *mental fatigue* correlated positively with age and disease duration. HRQOL was inversely correlated with *physical fatigue*, which was not associated with disease or participant characteristics. During 14 months of nusinersen treatment, fatigue measures remained mostly stable with a trend towards improvement in *reduced activity*, *general* and *physical fatigue*.

**Conclusion:**

Fatigue is a frequent and relevant complaint in adult SMA patients. Fatigue should be taken into consideration as additional outcome measure, but needs further evaluation in a larger patient cohort over a longer observation period.

## Introduction

Spinal muscular atrophy (SMA) is an inherited neuromuscular disorder, mainly caused by homozygous mutations in the *survival of motor neuron* (*SMN*) *1* gene on chromosome 5q [1]. The resulting lack of SMN protein leads to a degeneration of alpha motor neurons in spinal cord and brain stem. Patients suffer from progressive muscle weakness, scoliosis, contractures and respiratory insufficiency. Different SMA subtypes can be distinguished based on the age at symptom onset and the best motor milestones achieved. These phenotypes span a broad continuum, ranging from SMA type 0 with prenatal manifestation of hypotonia and respiratory failure at birth, to SMA type IV with development of muscle weakness in the second or third decade of life and normal life expectancy [2]. The dosage of *SMN2*, a neighboring nearly identical gene, has been identified as a potent modifier of SMA severity: a copy number of *SMN2* of ≥ 4 is highly predictive of achieving the ability to walk (SMA type III/IV) [3].

Since the broad approval of nusinersen, an antisense oligonucleotide which increases SMN protein production by modification of *SMN2* pre-mRNA splicing, a causative treatment option for SMA does exist. Pivotal trials were conducted in infants and children [4, 5]. Only recently, safety and feasibility of administration in older SMA patients has been demonstrated [6, 7]. First studies indicated beneficial treatment effects in adults and more data on long-term efficacy are soon to be expected [8–11]. It is controversial if classical motor function outcome measures are sensitive enough in patients with severely or very mildly impaired motor function, as floor and ceiling effects may occur [12, 13]. Further, motor function changes in severely affected patients may be small, but can have importance for the individual patient. Thus, facing the development of novel therapeutic options, alternative outcome measures besides motor function are needed.

Fatigue is a commonly reported symptom in neurological and especially neuromuscular disorders with an estimated prevalence of severe fatigue of 60% [14]. There is no exact definition of fatigue, but it is often described as “overwhelming sense of tiredness, lack of energy and feeling of exhaustion, mental, physical or both” [15]. Further, patients report an increasing sense of effort and a perceived mismatch between effort and actual performance or exhaustion. In contrast, the concept of fatigability (sometimes also referred to as “peripheral” fatigue) describes the decline in performance over time. Establishing a relationship between fatigue and fatigability has proven difficult [16].

Fatigue is believed to be a primary symptom of neurological disorders and not secondary to medication, laboratory abnormalities, mood or sleep disturbances. The prevalence of fatigue exceeds the—on the basis of age and disability—expected levels and, generally, fatigue does not correlate with the nature or severity of the underlying disease [14]. Regarding neuromuscular diseases, fatigue has so far been characterized in myasthenia gravis [17], post-polio syndrome [18, 19], congenital myopathies [20] and amyotrophic lateral sclerosis [21]. In SMA, fatigue was described early [22] and, from our clinical experience, is a frequent and burdening symptom. Surprisingly, there is little and sometimes even contradicting information on prevalence of and factors associated with fatigue in SMA [23–25]. Studies focusing on the several dimensions of fatigue and its impact on SMA patients, particularly in adults, are lacking.

In this study, we aimed to characterize perceived fatigue in adult SMA and identify associated factors. We further hypothesized that fatigue impacts quality of life and might be a suitable additional patient-reported outcome measure (PRO) in clinical trials or post-marketing surveillance.

## Methods

### Participants

SMA patients aged 18 years and above who received their first nusinersen administration were enrolled in a prospective longitudinal monocentric observational cohort study at the Department of Neurology of Hannover Medical School between April 2018 and October 2019. All had a genetically confirmed diagnosis of 5q-SMA (homozygous deletion of exon 7 or/and exon 8 of *SMN1*). All participants received nusinersen according to the approved scheme on days 0, 14, 28, 63 and every four months after completing the medication loading period. Our total SMA cohort consisted of 28 patients, 19 of whom received their first nusinersen administration at our facility and were, therefore, identified as potentially eligible. Complete baseline characteristics were available for 18 patients who were confirmed eligible and enrolled. 15 participants completed all questionnaires and assessments at the first nusinersen administration, one participant only completed MFI and not FSS. Reasons for refusal to fill in questionnaires were the impression of redundancy within questionnaires (*N* = 1) or not specified (*N* = 2). Follow-up data were collected during inpatient stays for nusinersen treatment. There was no loss to follow-up which minimized the risk of attrition bias. 14 months of follow-up data since therapy initiation were available for 14 participants, as two participants had not yet completed 14 months’ nusinersen administration at the time of data analysis and two had not completed the questionnaires after month 10. Reasons for non-completion of questionnaires at month 10 were specified as impression of redundancy (*N* = 1) and lack of time (*N* = 1). All collected data from all participants were included into the statistical analyzes.

Perceived fatigue and health-related quality of life were assessed using standardized questionnaires prior to and during treatment. Socio-demographic and disease characteristics including motor function were obtained from medical records or assessed at first administration.

### Fatigue measures

Participants completed the German Version of the Fatigue Severity Scale (FSS) and Multidimensional Fatigue Inventory (MFI) at day 0 (baseline), day 63 (month 2), month 6, month 10 and month 14. The FSS is one of the most frequently used unidimensional fatigue scales, which was initially developed for multiple sclerosis and systemic lupus erythemathodes [15, 26]. It is applicable in neuromuscular diseases and has been validated for SMA patients [20, 27]. It is a self-reported questionnaire and measures the impact of fatigue in the time frame of the “past week”. The scale consists of nine items with a seven point Likert-type scale. A mean score is calculated (ranging from one to seven), whereas a mean score of more than four is considered as abnormal fatigue and a score above five indicates severe fatigue [28, 29]. In case of a missing answer to an FSS item, the missing value was substituted with the mean of the remaining items [30].

As no other fatigue scales have been validated in SMA patients so far, we chose the MFI to measure the different dimensions of perceived fatigue. It is also a self-report instrument and contains 20 items which are categorized in five dimensions: *general fatigue*, *physical fatigue*, *mental fatigue*, *reduced motivation* and *reduced activity* [31]. The MFI measures how a patient felt “lately” on a five-point Likert-type scale. For each dimension, a total score is calculated, which can range from four to 20 while higher scores indicate increased fatigue. As fatigue measured by MFI is dependent on age and sex, cut-off values that indicate abnormal fatigue were defined as the respective 75th percentile of the German population according to Singer et al. (males: aged ≤ 39 years cut-off ≥ 8, 40—59 years cut-off ≥ 10, ≥ 60 years cut-off ≥ 13; females: aged ≤ 39 years cut-off ≥ 10, 40—59 years cut-off ≥ 11, ≥ 60 years cut-off ≥ 13) [32]. Missing answers were replaced with the mean of the respondent’s completed answers within the same subscale as previously described [33].

### Health-related quality of life measures (HRQOL)

Health state was measured with the German version of the EuroQol Five Dimension Five Level Scale (EQ-5D-5L), a standardized measure of HRQOL in adults [34, 35]. It captures five dimensions: mobility, self-care, usual activities, pain/discomfort and anxiety/depression. Each dimension is scaled in five levels of perceived problems (level one = no problems, level five = extreme problems) and together they result in a distinct health state (11111–55555). Those health states were converted into index values using the value set derived from the German reference sample according to the provider’s instructions [30, 36]. Additionally, the EQ-5D-5L comprises a vertical visual analog scale (VAS) scored from 0 to 100, capturing the “health state today” (higher scores indicate better HRQOL). The EQ-5D-5L has been validated in Europe and is frequently used in rare diseases, such as SMA [37, 38].

### Motor function measures

Motor function was assessed by professional physiotherapists using the Six-Minute Walk Test (6MWT), Hammersmith Functional Motor Scale Expanded (HFMSE) and Revised Upper Limb Module (RULM). The 6MWT assesses the submaximal aerobic capacity and has been found reliable and valid in ambulatory SMA patients [39]. The HFMSE is a disease-specific scale developed for SMA type II and III patients with ambulatory difficulties. It measures gross motor function using 33 items and calculating a sum score of up to 66 points (higher scores represent a better function) [40, 41]. The RULM, another disease-specific scale, measures function of upper extremities and performance in activities of daily living. It consists of 20 items with a maximum sum score of 37 points (higher scores indicate a better function) [42].

### Statistics

Statistical analysis was performed using IBM® Statistical Software Package of Social Science (SPSS®, Chicago, IL, USA) version 26. For all analyzes, significance levels were set at *p* < 0.05 (two-tailed). Descriptive statistics were calculated and depicted as percentage, mean and standard deviation or median and range. Data distribution was evaluated using Shapiro–Wilk and Kolmogorov–Smirnov tests. As not all data were distributed normally and considering the small number of participants, we chose non-parametric statistics. A Mann–Whitney *U* test for independent samples was used to determine differences between groups of dichotomous variables. All data were checked for outliers and, if present, analyses were repeated without them. Correlation was studied by means of Spearman rank correlation and, if suitable, linear regression was used to model the relationship between two metric variables. To evaluate outcome measures longitudinally, a two-way analysis of variance by ranks (Friedman’s test) for dependent samples was applied.

This study report was structured following the reporting guidelines to strengthening the Reporting of Observational Studies in Epidemiology (STROBE) [43].

## Results

### Participant characteristics

18 patients with SMA type II (*N* = 6), IIIa (*N* = 4), IIIb (*N* = 7) and IV (*N* = 1) were confirmed eligible and enrolled in the study. Ten participants had ≥ 4* SMN2* copies, whereof two participants reported symptom onset within 36 months after birth. Participant characteristics are summarized in Table 1. Mean age at first nusinersen administration was 37.1 years, mean duration of symptoms was 28.6 years. Half of the participants were ambulatory (*N* = 9), whereby ambulatory was defined as being able to walk without support for at least 10 meters [44]. Seven participants had scoliosis, four were dependent on intermittent non-invasive ventilation (NIV) and one had percutaneous endoscopic gastrostomy (PEG). Taken together, the study sample was severely impaired as reflected by mean HFMSE (27.2/66) and RULM (23.4/37).

**Table 1:** Socio-demographic and disease characteristics at baseline

	*N* (%)	Mean (SD)	Median (Range)
Female gender	7 (38.9)		
Age at therapy start (y)	18	37.1 (13.1)	34 (19–64)
Symptom duration (y)	18	28.6 (13.6)	30 (2–50)
SMA type			
Type II	6 (33.3)		
Type IIIa	4 (22.2)		
Type IIIb	7 (38.9)		
Type IV	1 (5.6)		
*SMN2* copy number			
< 4	8 (44.4)		
≥ 4	10 (55.6)		
Walking ability			
Non-ambulatory	9 (50.0)		
Ambulatory	9 (50.0)		
Scoliosis	7 (38.9)		
NIV	4 (22.2)		
PEG	1 (5.6)		
BMI < 18.5	5 (27.8)		
Education > 12 years	15 (83.3)		
Living with a partner	7 (38.9)		
6MWT distance (m)	9	345.4 (169)	415 (42–512)
HFMSE	18	27.2 (25.3)	19.5 (0–64)
RULM	18	23.4 (11.8)	23.5 (0–37)

Depicted are participant and disease characteristics as number (percentage), mean with standard deviation or median with range. The cut-off value for the education level was determined at 12 years as school attendance in most countries lasts up to 12 years. Remarkably, this sample displayed a high educational level with 83.3% reporting more than 12 years of formal education. Previous studies reported similar education levels in adult SMA patients [57]

*SMA* spinal muscular atrophy, *SMN2* *survival of motor neuron 2* gene, *NIV* non-invasive ventilation, *PEG* percutaneous endoscopic gastrostomy, *BMI* body mass index, *y* years, *6MWT* 6-Minutes Walk Test, *m* meter, *HFMSE* Hammersmith Functional Motor Scale Expanded, *RULM* Revised Upper Limb Module, *N* number, *SD* standard deviation

### Prevalence and dimensions of fatigue

At baseline, 53% of participants were classified as abnormally fatigued according to the FSS (FSS > 4), thereof six participants reported severe fatigue (FSS > 5). 53% agreed that fatigue was among their three most disabling symptoms according to item 8 of the FSS (item 8 ≥ 5). Considering MFI, age- and sex-adjusted prevalence of *general fatigue* was 75% in the total sample and all but one participant (94%) reported abnormal *physical fatigue*. Detailed prevalence rates and fatigue scores at baseline are shown in Table 2.

**Table 2:** Development of fatigue measures and prevalence rates during nusinersen treatment

	Baseline (*N* = 15)	Month 2 (*N* = 17)	Month 6 (*N* = 14)	Month 10 (*N* = 14)	Month 14 (*N* = 14)
FSS	4.31 (1.51), 53%	4.65 (1.44), 65%	3.93 (1.43), 43%	4.48 (1.35), 64%	3.87 (1.48), 43%
FSS item 8	3.87 (2.45), 53%	4.53 (2.12), 59%	3.29 (2.20), 43%	4.07 (1.94), 50%	3.86 (2.21), 43%

	Baseline (*N* = 16)	Month 2 (*N* = 17)	Month 6 (*N* = 15)	Month 10 (*N* = 14)	Month 14 (*N* = 14)
*General fatigue*	10.69 (3.44), 75%	10.94 (2.97), 82%	10.33 (2.77), 67%	11.46 (4.63), 86%	9.79 (3.97), 57%
*Mental fatigue*	8.62 (3.54), 56%	7.94 (3.21), 53%	7.93 (2.58), 53%	8.64 (3.84), 57%	9.71 (3.95), 64%
*Physical fatigue*	13.25 (4.07), 94%	13.24 (3.81), 82%	11.80 (3.99), 73%	12.43 (2.98), 86%	11.36 (3.61), 86%
*Reduced activity*	8.88 (3.14), 56%	10.00 (3.57), 71%	9.20 (3.84), 60%	10.00 (4.02), 50%	6.43 (2.34), 14%
*Reduced motivation*	6.69 (2.12), 31%	7.18 (3.19), 29%	6.93 (2.58), 7%	7.57 (3.63), 29%	8.00 (3.16), 50%

Data are expressed as mean score (SD), percentage of participants with clinically significant fatigue (prevalence). While mean scores of FSS, *general fatigue* and *physical fatigue* tended to decrease towards month 14, *mental fatigue* and* reduced motivation* slightly increased during treatment

*FSS* Fatigue Severity Scale, *MFI* Multidimensional Fatigue Inventory, *SD* standard deviation, *N* number

### Factors associated with fatigue

No significant differences between mean FSS or MFI scores were found regarding gender, living with a partner (yes versus (vs.) no), SMA type (types II/IIIa and types IIIb/IV were grouped together due to small sample sizes), scoliosis (yes vs. no), NIV (yes vs. no), BMI (< 18.5 vs. ≥ 18.5) and presence of pain/discomfort (EQ-5D-5L pain/discomfort dimension level one vs. levels two to five).

**Fig. 1:**
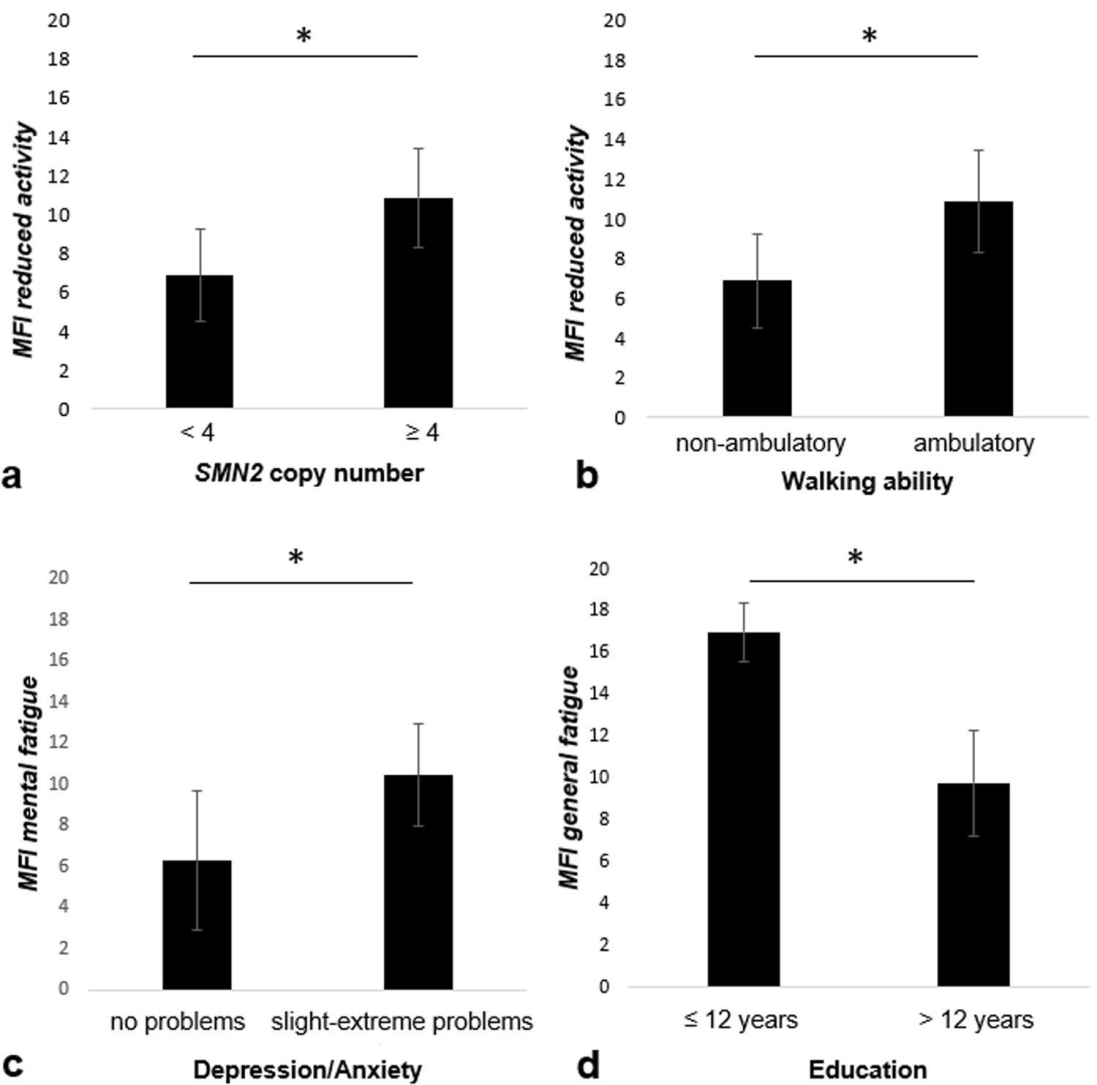
Association of fatigue with socio-demographic and disease characteristics. Mean score and standard deviation for each group are shown, *p < 0.05. **a** Higher scores in the *reduced activity* domain were reported by participants with ≥ 4 *SMN2* copies: ≥ 4 copies, mean = 10.88, SD = 2.53, *N* = 8; < 4 copies, mean = 6.88, SD = 2.36, *N* = 8 (*p* = 0.009, *Z* = − 2.545, Mann–Whitney *U* = 8.000). **b** Ambulatory participants reported higher scores in the *reduced activity* domain: ambulatory, mean = 10.88, SD = 2.53, *N* = 8; non-ambulatory, mean = 6.88, SD = 2.36, *N* = 8 (*p* = 0.005, *Z* = − 2.672, Mann–Whitney *U *= 6.500). **c** Higher scores in *mental fatigue* were reported by anxious or depressed participants: no problems with anxiety/depression, mean = 6.29, SD = 3.40, *N* = 7; slight—extreme problems with anxiety/depression, mean = 10.44, SD = 2.51, *N* = 9 (*p* = 0.023, *Z* = − 2.305, Mann–Whitney *U* = 10.000). **d** Participants with twelve or less years of formal education reported higher *general fatigue* scores: ≤ 12 years, mean = 17.00, SD = 1.414, *N* = 2; > 12 years, mean = 9.79, SD = 2.547, *N* = 14 (*p* = 0.017, *Z* = − 2.239, Mann–Whitney *U* = 0.000)

Participants with ≥ 4* SMN2* copies (and thus less severely affected) scored significantly higher in the MFI *reduced activity* domain compared to participants with less than four copies (*p* = 0.009). Similarly, ambulatory participants reported significantly higher levels of fatigue in the MFI *reduced activity* domain compared to non-ambulatory participants (*p* = 0.005) (Fig. 1a, b).

Regarding anxiety/depression (level one in the EQ-5D-5L anxiety/depression domain vs. levels two to five), significant differences were found for *mental fatigue*: participants without anxiety/depression reported less *mental fatigue* (*p* = 0.023) compared to those who had slight to extreme anxiety/depression. Further, participants with higher education levels (> 12 years of school attendance) reported significantly lower *general fatigue* with the limitation that in our sample, there were only two participants with ≤ 12 years of school attendance (*p* = 0.017) (Fig. 1c, d).

**Fig. 2:**
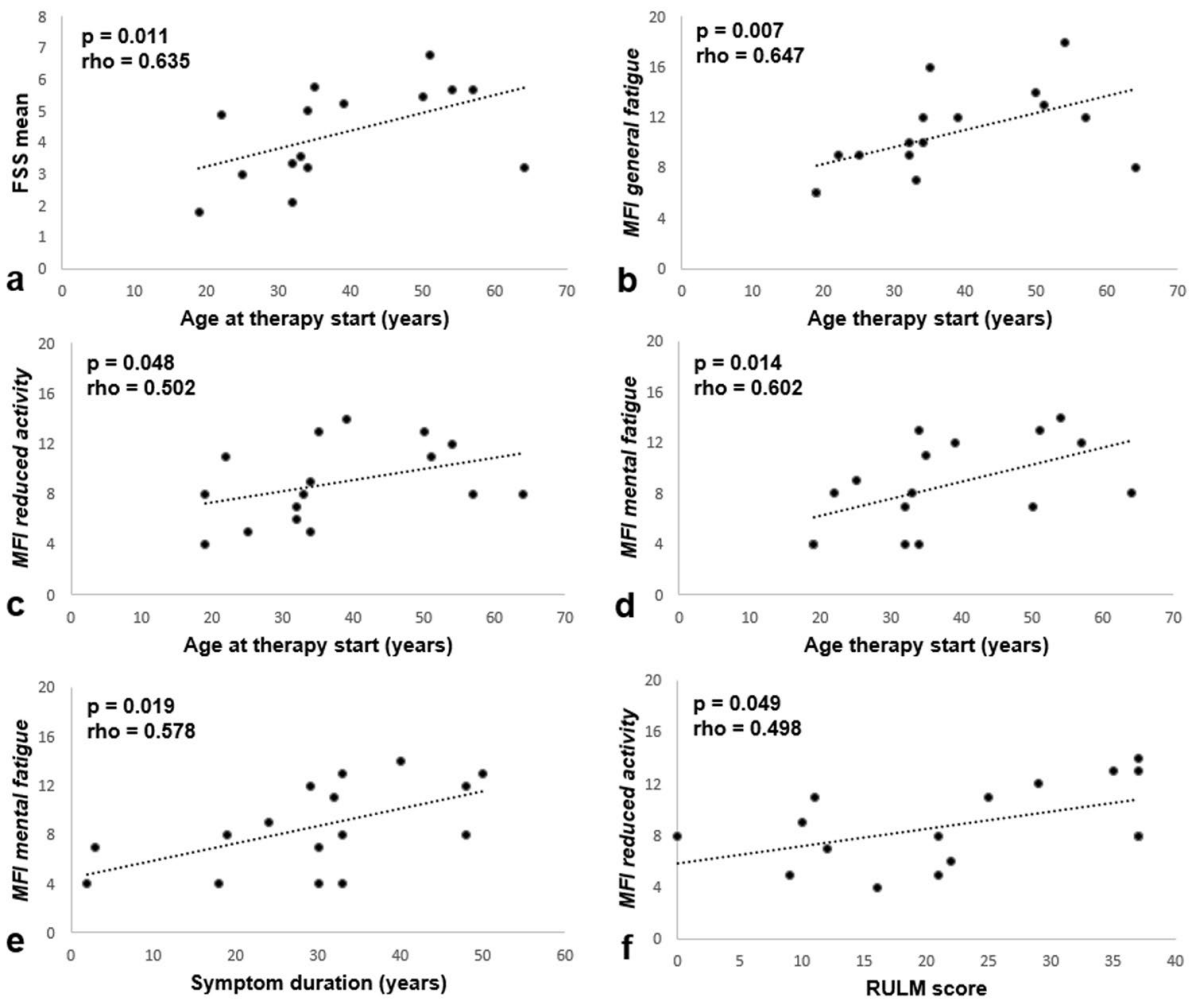
Correlation of fatigue measures, age at therapy start, symptom duration and motor function. Adjustment curves of linear regression analyzes are shown. Each dot represents one participant. **a + b** Fatigue Severity Scale (FSS) mean score (Spearman-Rho = 0.635, *p* = 0.011, *R*^2^ = 0.251, *N* = 15) and *general fatigue* assessed via Multidimensional Fatigue Inventory (MFI) (Spearman-Rho = 0.647, *p* = 0.007, *R*^2^ = 0.289, *N* = 16) correlated positively with age at therapy start. **c** The *reduced activity* domain of the MFI correlated positively with age at therapy start (Spearman-Rho = 0.502, *p* = 0.048, *R*^2^ = 0.151, *N* = 16). **d + e** *Mental fatigue* correlated positively with age at therapy start (Spearman-Rho = 0.602, *p* = 0.014, *R*^2^ = 0.281, *N* = 16) and symptom duration (Spearmen-Rho = 0.578, *p* = 0.019, *R*^2^ = 0.319, *N* = 18). **f** Participants with higher RULM scores reported more fatigue in the *reduced activity* domain of the MFI (Spearman-Rho = 0.498, *p* = 0.049, *R*^2^ = 0.264, *N* = 16)

Correlation analysis and linear regression of fatigue measures and age at therapy start revealed a significant relationship of the FSS mean score and three MFI subdomains: Participants who were older at the time of treatment initiation reported more *general* and *mental fatigue* as well as higher scores on the MFI *reduced activity* domain (FSS: *p* = 0.011, *general fatigue*: *p* = 0.007, *mental fatigue*: *p* = 0.014, reduced activit*y*: *p* = 0.048) (Fig. 2a–d). Further, a significant positive correlation was observed between symptom duration and *mental fatigue* (*p* = 0.019) (Fig. 2e). Regarding motor function, we found a positive correlation between RULM scores and the MFI *reduced activity* domain: participants with better upper limb motor function reported higher fatigue (*p* = 0.049) (Fig. 2f), whereas HFMSE did not correlate significantly with fatigue measures.

Correlations between FSS and MFI dimensions were as follows: FSS correlated best with *general fatigue*, but did not correlate significantly with *physical fatigue* or *reduced motivation*. *Mental fatigue* correlated significantly with all other scores and *general fatigue* correlated significantly with all scores but *reduced motivation*. *Physical fatigue* did only correlate significantly with *general* and *mental fatigue*, but not with *reduced activity* or *reduced motivation* (data not shown).

### Factors associated with health-related quality of life (HRQOL)

HRQOL represented by EQ-5D-5L index values was not normally distributed in our sample (mean = 0.46, SD = 0.37; median = 0.18, range = 0.03–1.00), but showed a cluster formation: one cluster with an index value below 0.2 (*N* = 9) and another cluster with an index value above 0.68 (*N* = 7). Accordingly, we presumed that index values in our cohort were not representative for HRQOL in 5q-SMA and, thus, excluded the index values from further analysis. In contrast, EQ-5D-5L VAS values were distributed normally with a mean value of 57.06 (SD = 19.61) and median value of 60 (range 25–100).

**Fig. 3:**
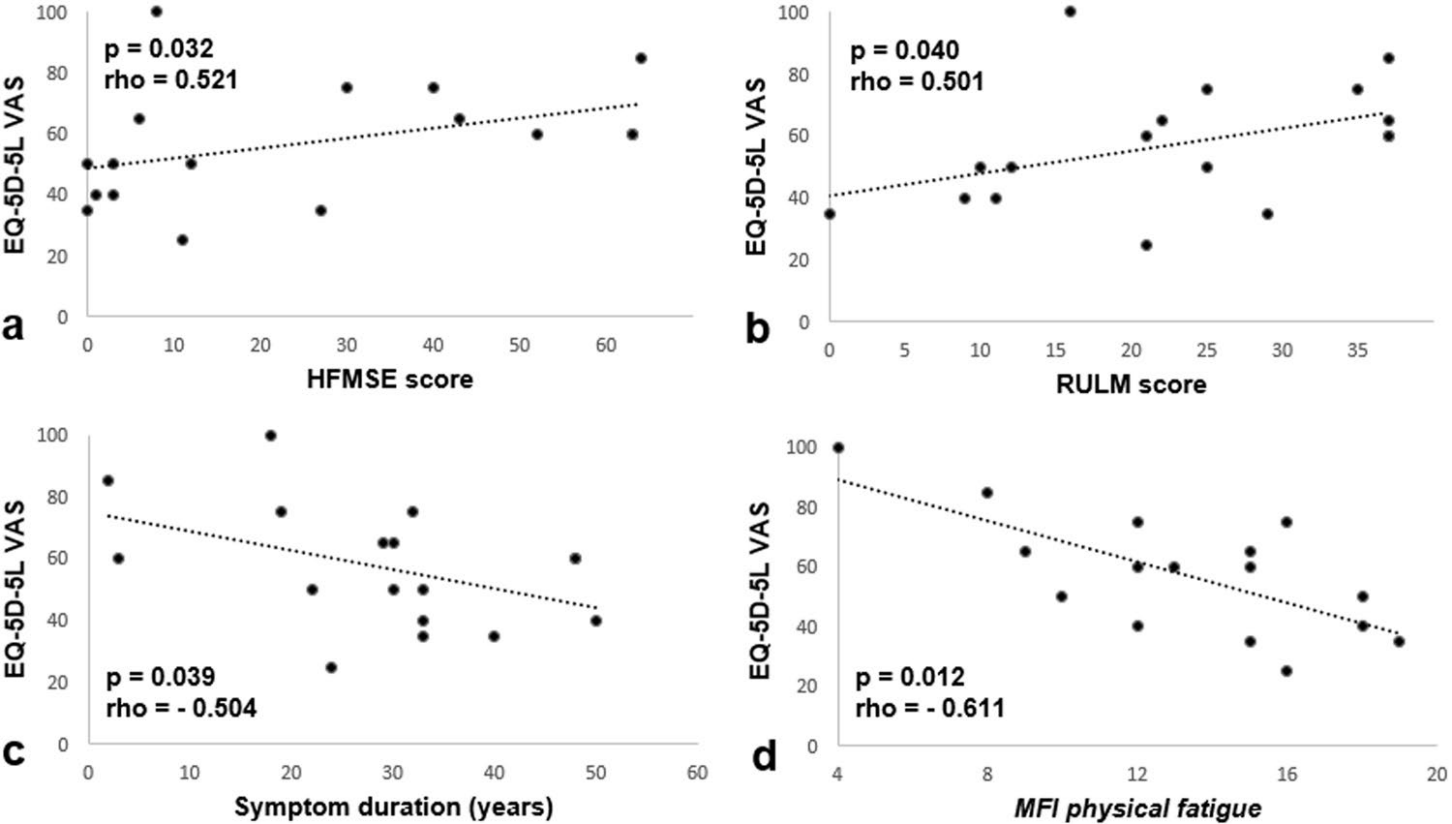
Factors associated with health-related quality of life (HRQOL). Adjustment curves of linear regression analyzes are shown. Each dot represents one participant. **a–c** HRQOL via EQ-5D-5L VAS correlated positively with HFMSE (Spearman-Rho = 0.521, *p* = 0.032, *R*^2^ = 0.172, *N* = 17) and RULM scores (Spearman-Rho = 0.501, *p* = 0.040, *N* = 17, *R*^2^ = 0.181). We further observed a negative correlation of HRQOL via EQ-5D-5L VAS with symptom duration (Spearman-Rho = − 0.504, *p* = 0.039, *R*^2^ = 0.191, *N* = 17). **d** The adjustment curve of linear regression analysis of HRQOL and *physical fatigue* assessed via Multidimensional Fatigue Inventory (MFI) showed a significant negative correlation (Spearman-Rho = − 0.611, *p* = 0.012, *R*^2^ = 0.487, *N* = 16)

No significant differences in HRQOL measured by EQ-5D-5L VAS could be detected for the variables gender, BMI, education level, relationship status, prevalence of pain/discomfort or depression/anxiety, SMA type, *SMN2* copy number, scoliosis, walking ability, NIV or age at therapy onset. Correlation analysis, on the other hand, revealed a negative relation of HRQOL with symptom duration (*p* = 0.039) and a positive relation with HFMSE and RULM scores (*p* = 0.032 and *p* = 0.04) (Fig. 3a–c). Further, correlation analysis of fatigue measures and EQ-5D-5L VAS showed a significant linear relationship for *physical fatigue* (*p* = 0.012): participants who reported higher *physical fatigue* scores presented with lower health-related quality of life (Fig. 3d).

### Development of perceived fatigue during nusinersen treatment

**Fig. 4:**
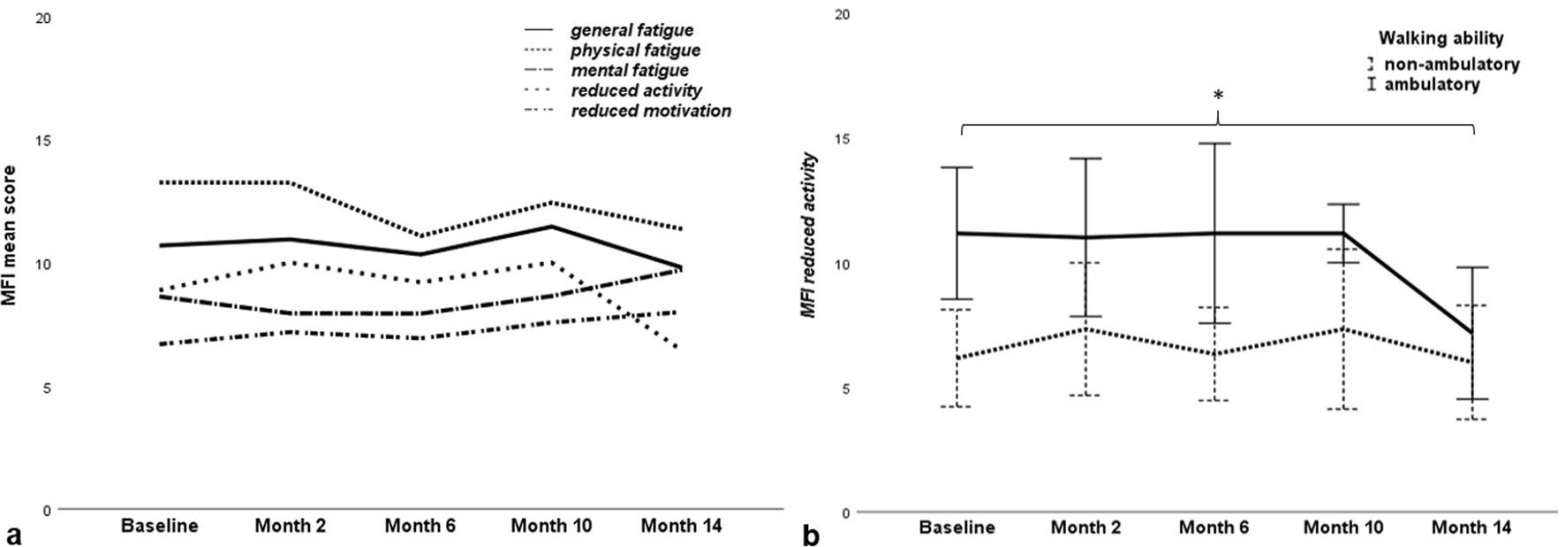
Development of fatigue under nusinersen treatment. **a** Mean scores of the five MFI dimensions are depicted during 14 months of nusinersen treatment. While *general fatigue*, *physical fatigue* and *reduced activity* tended to decline, *mental fatigue* and *reduced motivation* showed a tendency to increase towards month 14. **b** Mean scores and standard deviations of *reduced activity* are depicted during nusinersen treatment. The dashed line represents the non-ambulatory participants, the solid line the ambulatory participants. There was a significant decline of *reduced activity* in ambulatory participants at month 14 [*p* = 0.018, *N* = 6, test statistic = 11.930, degrees of freedom = 4, *p* (pairwise comparison between baseline and month 14) = 0.026], *p < 0.05

By applying Friedman’s test, no significant differences between fatigue scores at different times during nusinersen treatment were detected (Table 2). Mean scores for item 8 of the FSS decreased during treatment (*p* = 0.026, test statistic = 11.038, degrees of freedom = 4, *N* = 12), but pairwise comparison did not reveal any significant changes. There was a trend towards a decrease in fatigue during treatment, with the lowest score after 14 months for FSS, *general* and *physical fatigue*. Mean scores for *reduced activity* also decreased towards month 14 (*p* = 0.024, test statistic = 11.262, degrees of freedom = 4, *N* = 12), but significance was also slightly missed after Bonferroni correction in pairwise comparison. *Mental fatigue* and *reduced motivation* in contrast showed a tendency to increase towards month 14 (Fig. 4a) (Table 2). In subgroup analysis, participants with ≥ 4 *SMN2* copies and ambulatory participants showed a significant decline in the *reduced activity* domain from baseline to month 14 (*p* = 0.026) (Fig. 4b).

As mean scores represent raw data and prevalence rates via MFI were adjusted for age and gender, we additionally evaluated the change of prevalence rates. The prevalence of *physical fatigue* and *reduced motivation* showed a tendency to decrease during nusinersen treatment with lowest scores at month 6. After month 6, *reduced motivation* tended to increase along with *mental fatigue*. The prevalence of *general fatigue* fluctuated and prevalence of *reduced activity* increased within the first two treatment months followed by a remarkable decrease afterwards. According to FSS item 8 (“Fatigue is among my three most disabling symptoms”), fatigue was a relevant complaint in 43% of the participants at month 14, compared to 53% at baseline (Table 2).

### Fatigue compared to motor function development

To evaluate the suitability of fatigue as an additional outcome measure, we further investigated fatigue scores compared to the development of motor function under nusinersen therapy. Participants were dichotomized into two groups: participants whose RULM/HFMSE scores increased during 14 months of treatment were compared to participants with deteriorated or unchanged RULM/HFMSE scores, as an improvement of motor functions is not observed in the natural course of SMA and most likely is a therapy effect. Mean changes in RULM scores for these subgroups were + 3 (SD = 2, *N* = 3) and ± 0 (*N* = 11). None of the participants showed a decrease of RULM score. Initial fatigue scores at baseline did not differ significantly, but there was a trend that participants who showed a RULM score increase reported less fatigue, especially less *physical fatigue*. At months 2, 6 and 10, this trend became statistically significant for *physical fatigue* (month 2: *p* = 0.028, *N* = 13, Z = − 2.133, Mann–Whitney *U* = 2.500; month 6: *p* = 0.014, *N* = 13, *Z* = − 2.35, Mann–Whitney *U* = 1.500; month 10: *p* = 0.036, *N* = 12, *Z* = − 2.16, Mann–Whitney *U* = 2.000). *Mental fatigue* was reported significantly less by participants with improved RULM scores at month 2 (*p* = 0.007, *N* = 13, *Z* = − 2.58, Mann–Whitney *U* = 0.000) and month 6 (*p* = 0.007, *N* = 13, *Z* = − 2.62, Mann–Whitney *U* = 0.000). At month 14, no significant differences between RULM groups were observed. However, these findings have to be interpreted with care due to the small number of participants in the group with improved RULM score (*N* = 3). Mean changes in HFMSE scores were + 3.13 (SD = 4.05, *N* = 8) and − 1.43 (SD = 1.40, *N* = 7), accordingly. There were no significant differences in fatigue scores between participants with increased HFMSE during treatment and those with deteriorated/stable HFMSE at any time.

## Discussion

In this study, adult 5q-SMA patients frequently reported fatigue as one of their most disabling symptoms. *Physical fatigue* followed by *general fatigue* and *reduced activity* were identified as the most relevant dimensions. We found that socio-demographic factors were associated with *general* and *mental fatigue*, while disease characteristics and motor function were associated with *reduced activity*. *Physical fatigue* correlated negatively with HRQOL, but showed no association with participant or disease characteristics. Interestingly, after more than one year of nusinersen treatment, less participants tended to report fatigue as one of their most disabling symptoms and there was a trend towards a decrease of* physical* and *general fatigue* as well as *reduced activity*. In the subgroup of ambulatory participants, *reduced activity* significantly improved at month 14. Participants with increasing RULM scores under therapy reported significantly less *physical fatigue* between month 2 and 10 of treatment as well less *mental fatigue* between month 2 and 6 compared to participants with unchanged RULM scores.

To measure multiple dimensions of fatigue in our study cohort, we made use of two independent questionnaires. While the FSS has been validated in SMA patients [20], this is—to our knowledge—the first study involving the MFI. The mean FSS score at baseline was 4.31 which is consistent with the recent report of Kizina et al. who found a mean score of 4.61 in adult SMA patients awaiting nusinersen treatment [45]. The discrepancy between the prevalence rates of 53% (FSS) and 75% (MFI *general fatigue*) might be explained by the nature of the assessment instruments: while the FSS measures the impact of fatigue on functioning, the MFI primarily measures phenomenology and severity of fatigue. Further and most importantly, prevalence was reported as raw data for the FSS. Meanwhile, for MFI, age- and gender-adjusted cut-off values were used as suggested by Singer et al. [32]. As fatigue is dependent on age and gender [46, 47], which in part was also applicable in our cohort, prevalence estimation might be more accurate using those age- and gender-adjusted cut-off values and the estimated prevalence of 75% via MFI seems to be more precise. This is in line with the previously reported prevalence of fatigue of 81–100% in spinobulbar muscular atrophy (SBMA) and SMA patients assessed via FSS and PedsQL™ Multidimensional Fatigue Scale [24, 25]. Regarding the development in fatigue over the course of 14 months, both instruments depict a similar relative change for *general fatigue*, which indicates their suitability to measure the same construct in the long term.

In the present study, 94% of the participants reported abnormal *physical fatigue*, which indicated its importance in adult SMA. As patients with disabling motor neuron diseases are not able to perform exercise in the classical sense (e.g. running or weight lifting), they tend to report less activity determined fatigue [48], which is assessed by most fatigue instruments (for example FSS item 2: “Exercise brings on my fatigue”). The MFI, on the other hand, addresses the perceived physical condition in general rather than fatigue following exercise and might, therefore, be more sensitive in SMA patients (for example item 2: “Physically I feel only able to do a little” or item 14: “Physically I feel I am in a bad condition”). In accordance with previous reports regarding fatigue in general [14, 21, 25], *physical fatigue* did not correlate with disease severity or motor function in our cohort. Nevertheless, it was associated with deteriorated HRQOL as previously described for fatigue in motor neuron diseases [49], while HRQOL itself correlated positively with measures of motor function. Further, *physical fatigue* showed a tendency to decrease under treatment and was temporarily significantly lower in the subgroup of participants with improved RULM scores. Therefore, *physical fatigue* appears to be a symptom independent of motor function which has impact on HRQOL and a further evaluation as PRO seems worthwhile. In contrast, the FSS did not correlate with HRQOL or resemble motor improvements in our cohort and seems to be less suitable as outcome measure in adult SMA patients in the short term.

Next, we found that older participants and participants with < 12 years of education suffered more extensively from *general fatigue* according to FSS and MFI, which is in line with findings in the general population and might be an effect independent of SMA [46, 47]. Though, the correlation between education and fatigue should be interpreted with care regarding the size of our cohort (≤ 12 years of education: *N* = 2). *Mental fatigue* scores were higher in participants with depression/anxiety, longer symptom duration and higher age at therapy start. These effects have also been shown for *general* and *mental fatigue* in other neurological diseases, such as multiple sclerosis and Parkinson’s disease [15, 50, 51]. Surprisingly, we found no effect of discomfort/pain on perceived fatigue as it has been shown for various conditions [33, 52, 53]. A possible explanation might be that pain is not a primary symptom in SMA. *Reduced motivation* seemed to be less of a problem in SMA patients with a mean score of 6.69, which is comparable to general population [54].

In contrast to the other fatigue dimensions, subgroup and correlation analysis of *reduced activity* revealed significantly higher scores in less severely impaired participants (≥ 4 *SMN2* copies, ambulatory, higher RULM scores) and after 14 months of nusinersen treatment *reduced activity* significantly improved within this subgroup. Nonetheless, especially the domain of *reduced activity* might be sensitive to SMA severity. Patients with a higher *SMN2* copy number might experience a greater mismatch between their own reached best activity level (before disease onset) and their actual capacities/activities compared to those with less *SMN2* copies who, e.g. never learned to walk. Additionally, patients with milder disease severity and later disease onset might be less adapted to their impairment (fewer auxiliary devices and personnel) and, therefore, experience more limitations in their activities. We assume that *reduced activity* might not reflect perceived fatigue in our cohort but rather a phenomenon independent of fatigue as it is associated with disease severity but not with quality of life.

Recently, fatigability in ambulatory children and adolescent SMA patients (measured by physiotherapists as distance walked in the 6MWT) has shown clinically meaningful improvement under nusinersen treatment [55]. As opposed to this study, we used assessments of perceived fatigue feasible for all patients independent of motor function impairment. Overall, the most prevalent fatigue dimensions tended to decrease during 14 months of nusinersen treatment. As there are no data available on the natural course of fatigue among SMA patients, the interpretation of our results remains difficult. However, fewer participants tended to rate fatigue as one of their most disabling symptoms at month 14. *Reduced activity* showed a tendency to increase intermittently during treatment with nusinersen and reached its maximum at month 2. This might reflect the increased treatment burden during the loading period. During the maintenance period, *reduced activity* levels remarkably declined. Temporary improvements in *mental fatigue* and *reduced motivation*, on the other hand, might have been biased by positive pre-treatment expectations, while increased scores after ten and 14 months could reflect treatment burden or reluctance. Prevalence of *general fatigue* (via MFI and FSS) tended to be decreased at month 14 compared to baseline. In general, we observed a fluctuation of fatigue scores and prevalence rates in the longitudinal assessment. This underlines the exploratory character of our study. An investigation over a longer time period seems to be unavoidable to detect relevant treatment effects because of the slowly progressive disease course in the investigated patient cohort. Longer observation and treatment duration are also needed to assess long-term efficacy and sustainability of effects.

This study has further limitations. Primarily, it is limited by the relatively small sample size of 18 participants due to which we might not have been able to demonstrate significant effects of covariates or treatment. To improve generalizability, analyses of larger samples including SMA type I patients should be initiated through national and international cooperation. Due to the absence of a control group, we can only calculate absolute effect measures. However, withholding nusinersen treatment from SMA patients is ethically not justifiable and there seem to be only few SMA patients who decline nusinersen treatment. Further, not all conceivable covariates which might influence perceived fatigue, such as sleep disturbances, dyspnea, hemoglobin levels or employment status, were recorded [33, 56]. Finally, we have to take the possibility of selection bias into account as we cannot be totally sure that the participating SMA patients are representative of all SMA patients. Patients with longer symptom duration might not have come forward to seek treatment, which might have led to an underestimation of fatigue prevalence.

In conclusion, we characterized perceived fatigue in adult SMA patients and identified it as a frequent and burdening symptom. While disease severity and motor function were associated with *reduced activity*, socio-demographic factors as age, education and depression influenced *general* and *mental fatigue*. *Physical fatigue* as most prevalent dimension of fatigue correlated negatively with HRQOL, but was independent of participant and disease characteristics. Fatigue measures fluctuated during 14 months of nusinersen treatment with a trend towards reduced* physical* and *general fatigue* and improved *reduced activity*. Perceived fatigue, especially *physical fatigue* and *reduced activity*, appears to be a suitable PRO additionally to motor function outcome measures, as it (1) is frequent in SMA, (2) is a complaint relevant to patients which correlates with quality of life, (3) can be assessed easily and (4) might be more sensitive in detecting subjective improvement/worsening in more severely affected patients than motor function measures. Larger patient cohorts and longer observation periods are needed to further address this suggestion.

## Data Availability

De-identified data will be shared on reasonable request with any qualified investigator.
